# Dr. Beddoes, on the Anti-Venereal Power of Acids

**Published:** 1801-01

**Authors:** Thomas Beddoes, C. Forbes

**Affiliations:** Bombay


GENTLEMEN,

SINCE the publication of thoſe obſervations, which I have ventured to call demonſtrative of the anti-venereal power of acids, I have received further teſtimonies. I encloſe a ſpecimen.

I have made ſome progreſs in a collection towards defraying the expence of a ſet of public experiments. Mr. Pearſon thinks with me, that a ſeparate eſtabliſhment is beſt ſuited to the deciſion of this queſtion. Among other regulations, it ſeems neceſſary for ſatisfying the public mind, that a perſon of unſuſpected probity ſhould be fixed upon to adminiſter the acids; that this perſon ſhould ſwear to the fidelity of his reports; and that the nurſe and patients ſhould ſwear, the one not to ſupply, or cauſe to be ſupplied, any article not directed; the other, not to take any ſuch articles.

It is not merely for the ſake of bringing our controverſy, important as it is, to an iſſue, that ſuch a plan is deſirable; the contradictory reports place before our eyes the exiſtence of a moſt criminal levity in obſerving a diſregard to truth in reporting. What human being, who conſiders that he himſelf, and thoſe he moſt values, are one day deſtined to become the ſubjects of medicinal proceſſes, but muſt tremble when he perceives that there muſt be, at the preſent time, in this enlightened country, a conſiderable number of reputable medical practitioners, incompetent to the deciſion of one of the eaſieſt and moſt palpable problems belonging to their art; who cannot, or will not uſe their ſenſes with a little coolneſs, patience, and impartiality? When ſuch a perſon is to be treated for an obſcure internal diſeaſe, in what hands ſhall he deem himſelf tolerably ſecure?

It appears from an hundred examples, that any man may publiſh almoſt any thing concerning any remedy, with ſome chance of a lucrative reputation, and at ſcarce any hazard of ruinous diſgrace. Not only, therefore, will a public ſcrutiny expoſe that party, which, in the diſpute concerning the acids, is chargeable with ſuch portentous incapacity; but it will ſerve as a wholeſome leſſon to thoſe who are ready to diſcharge their crudities upon the public.

For my own part, I feel aſſured that the inquiry will confirm the favourable atteſtations. And there are two arguments ariſing from a comprehenſive view of the documents on both ſides, which muſt ſtrike impartial judges. 1. The affirmative caſes were very open to inſpection; and at Plymouth, Woolwich, wich, and in India, they were actually inspected by a number of medical men, in no respects biassed in favour of acids. 2. Among the assertors are persons equally versed in medical observation, and in experiments on inanimate matter; an exercise which habituates the eye to examine with accuracy, and the mind to reason strictly, and which ought to be made an indispensable requisite in medical education.

So much having been said in Europe on the subject of relapses, the East India practitioners have been induced to attend particularly to this objection. And Dr. Helenus Scott, in a letter too late for my last pamphlet, thus states the result.

"With regard to relapses, when recent cases have been cured by the nitric acid, I believe they occur less frequently than after mercury. Mr. Stuart, Dr. Keir, Mr. Macgregor, and myself, have cured by it, within these eighteen months, above one hundred patients with primary symptoms, one only of whom has relapsed. This man, a patient of Mr. M'Gregor, came back to him with a small chancre on the prepuce, but it is doubtful if this did not arise from a fresh infection. No practitioner surely will assert that so great a number would have continued well, who in this country had been treated by mercury."

I am glad of an opportunity to mention that my pamphlet is much disfigured by errors of the press. In many places of the preliminary discourse, in spite of my corrections, the London printer has made me write strange English. There are few places, however, where the sense cannot be made out; and I hope by this time, that all the copies are provided with a list of errata. I only beg leave to mention here, that at p. 51, l. 21, *citric* acid should be read in place of *nitric*. I am,

GENTLEMEN,

Respectfully, your's,

*December* 12, 1800. THOMAS BEDDOES.

---

### To Dr. KIER, from CHARLES FORBES, Esq.

My dear Sir,

Agreeably to your desire, I now send you the particulars of the cure effected by Dr. Scott on one of our servants, by the use of the nitric bath.

About a twelvemonth ago, the servant alluded to (a native boy of 16 or 17 years of age) was seized with violent pains all over his body, but particularly in his head and loins, which were most severe in the night time, and entirely deprived him of rest, except what he procured with the assistance of tobacco or opium, he foon became reduced to a fkeleton, and fo ill that he had nearly loft the ufe of one of his legs and thigh, the mufcles of which were fo much contracted that he could only put his toes to the ground, and his fkin was covered with white fcabs or fcales. Hitherto he had perfifted in denying that he was ever infected with the venereal difeafe, and attributed the pains in his bones, and contraction in his leg and thigh, to a ftroke of the land wind, which was corroborated by the opinion of the other fervants; he now confeffed, however, that he had caught the venereal difeafe about a year before, which he defcribed to have been a running and ulcers, and that he removed thofe fymptoms by drinking milk, which had been recommended to him by one of his friends, but that he took no mercury nor any other medicine. On defcribing the boy's cafe to Dr. Scott, he was fo good as to vifit him, and recommended the nitric bath, which was immediately commenced in the ufual way, at firft twice daily, and about half an hour at a time; but it was remarkable, that in the courfe of a week, the boy found himfelf fo much relieved, that he repeated the bath half a dozen times in the day of his own accord, and at length would frequently fit two or three hours at a time up to the neck in it, until his fkin became fo fore that he could hardly bear to put on his clothes. His head-achs had now left him, he refted well, and only complained of the pain in his loins, which, by a moderate ufe of the bath, was alfo removed in the courfe of about a month from the time he firft began it, and he gradually recovered the ufe of his leg. He is now in perfect health, and has had no return of his complaints. He never took mercury, but his mouth was confiderably affected by the ufe of the nitric bath.

I take this opportunity of mentioning two other inftances of the efficacy of the nitric acid, which have come within my immediate obfervation. The firft is that of John Hibernia, a Caffree, aged about forty, who was fervant to the late Colonel Nugent, and whom Mr. Forbes put under Dr. Scott's care about two years ago; he was afflicted with venereal pains in his bones, for which he had been repeatedly falivated by mercury, but received only a temporary relief from it, for when the effects of the mercury ceafed, his pains returned. Dr. Scott adminiftered the nitric acid internally, and in the courfe of a few weeks compleatly cured him. I lately met him on the road, and on queftioning him refpecting his health, he informed me that he was perfectly well, and had been fo for a twelvemonth paft.

The fecond inftance alluded to, is that of an officer of one of our fhips, for whom I requefted Mr. Boag's advice. Having accidentally fcratched his finger, it turned into an ugly fore, which gave him exceffive pain, and although timely applications were made, it feemed to defy them all, and at length put on fo putrid an apperaance that Mr. Boag thought the bone was affected, and that the lofs of the finger muft enfue. He now recommended a trial of the nitric acid upon it; and by bathing the finger in it, three or four times a day, as ftrong as it could be borne, the fore was foon healed up, and the finger got perfectly well.

I remain, your's,

*Bombay, Jan.* 1800. C. FORBES.

---

